# Supplementary material for: Education differentiates cognitive performance and resting state fMRI connectivity in healthy aging
Source: Front Aging Neurosci. 2023 May 24;15:1168576. doi: 10.3389/fnagi.2023.1168576 (PMC10244540; doi:10.3389/fnagi.2023.1168576)
Supplement: Supplementary file 1 [file Data_Sheet_1.doc]

**Supplementary Material**

***Education differentiates cognitive performance and resting state fMRI connectivity in healthy aging***

Sonia Montemurro, PhD ^1†^, Nicola Filippini, PhD ^1†^, Giulio Ferrazzi, PhD ^1,2^, Dante Mantini, PhD ^3^, Giorgio Arcara, PhD ^1 *^, Marco Marino, PhD ^3,4 *^

^1^IRCCS San Camillo Hospital, Venice, Italy

^2^Philips Healthcare, Milan, Italy

^3^Movement Control and Neuroplasticity Research Group, KU Leuven, Belgium

^4^Department of General Psychology, University of Padua, Italy

^†^these authors share first authorship

^*^these authors share last authorship

**VBM analysis to show age-related gray matter difference**

**
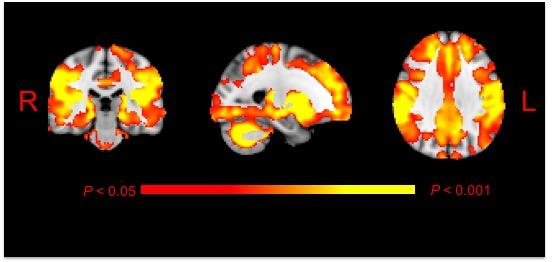
**

**Figure S1. Age-related gray matter (GM) differences. In yellow/red, the brain** regions where older participants had a reduction in GM volume compared with younger participants using a whole-brain VBM analysis approach (p < 0.05 corrected for multiple comparisons).

**Correlation analysis among RSNs and cognitive variables selected by the LEMON dataset**

|  |  | Immediate  Memory | | Delayed Memory | | Phonemic  Fluency | | Semantic  Fluency | | Vocabulary | |
| --- | --- | --- | --- | --- | --- | --- | --- | --- | --- | --- | --- |
|  | RSNs | rho | p-val | rho | p-val | rho | p-val | rho | p-val | rho | p-val |
| Y  (137) | VMN | 0.08 | 0.50 | 0.05 | 0.53 | -0.06 | 0.48 | 0.03 | 0.68 | -0.03 | 0.72 |
|  | DAN | 0.03 | 0.65 | 0.04 | 0.58 | 0.01 | 0.88 | 0.05 | 0.51 | -0.03 | 0.69 |
|  | DMN | 0.05 | 0.33 | 0.05 | 0.54 | -0.01 | 0.89 | -0.01 | 0.95 | -0.13 | 0.13 |
| O  (60) | VMN | -0.13 | 0.33 | -0.07 | 0.56 | -0.03 | 0.81 | 0.15 | 0.24 | -0.07 | 0.57 |
|  | DAN | -0.23 | 0.08 | -0.18 | 0.16 | 0.05 | 0.69 | 0.04 | 0.76 | -0.05 | 0.69 |
|  | DMN | **-0.30** | **0.02** | **-0.30** | **0.02** | -0.10 | 0.43 | 0.03 | 0.81 | 0.01 | 0.89 |
| OH  (30) | VMN | -0.31 | 0.11 | -0.28 | 0.14 | 0.05 | 0.79 | -0.06 | 0.73 | 0.13 | 0.51 |
|  | DAN | -0.35 | 0.06 | -0.31 | 0.11 | -0.15 | 0.42 | -0.13 | 0.50 | 0.05 | 0.77 |
|  | DMN | -0.35 | 0.06 | -0.34 | 0.04 | 0.17 | 0.38 | -0.02 | 0.90 | 0.06 | 0.74 |
| OL  (30) | VMN | 0.18 | 0.36 | 0.24 | 0.22 | -0.14 | 0.43 | 0.37 | 0.05 | -0.28 | 0.15 |
|  | DAN | -0.06 | 0.73 | -0.05 | 0.79 | -0.04 | 0.81 | 0.11 | 0.60 | -0.12 | 0.54 |
|  | DMN | -0.03 | 0.86 | -0.01 | 0.99 | 0.29 | 0.13 | 0.29 | 0.13 | -0.13 | 0.52 |

**Table S1. Correlation analyses and related p-values. fMRI Resting State Networks (RSNs) and cognitive variables in young adults, and older adults grouped by education.** Cognitive variables: Immediate Memory, Delayed Memory, Phonemic Fluency, Semantic Fluency, and Vocabulary values, and functional connectivity values that showed group-difference in the ANCOVA analyses, VMN, DAN, and the DMN. Pearson correlation coefficients and their significance were calculated separately for each group, including young (Y), old (O), old with higher level of education (OH), and old with lower level of education (OL). Bold values indicate correlations with *p* < 0.05.
